# Reliability of the NICMAN Scale: An Instrument to Assess the Quality of Acupuncture Administered in Clinical Trials

**DOI:** 10.1155/2017/5694083

**Published:** 2017-06-12

**Authors:** Caroline A. Smith, Christopher J. Zaslawski, Suzanne Cochrane, Xiaoshu Zhu, Zhen Zheng, Bertrand Loyeung, Peter C. Meier, Sean Walsh, Charlie Changli Xue, Anthony L. Zhang, Paul P. Fahey, Alan Bensoussan

**Affiliations:** ^1^National Institute of Complementary Medicine, Western Sydney University, Penrith, NSW, Australia; ^2^School of Life Sciences, The University of Technology Sydney, Sydney, NSW, Australia; ^3^School of Health and Biomedical Sciences, RMIT, Melbourne, VIC, Australia; ^4^Faculty of Science, The University of Technology Sydney, Sydney, NSW, Australia; ^5^School of Science and Health, Western Sydney University, Penrith, NSW 2751, Australia

## Abstract

**Background:**

The aim of this study was to examine the reliability of a scale to assess the methodological quality of acupuncture administered in clinical research.

**Methods:**

We invited 36 acupuncture researchers and postgraduate students to participate in the study. Firstly, participants rated two articles using the scale. Following this initial stage, modifications were made to scale items and the exercise was repeated. Interrater reliability was assessed for individual items using the Fleiss kappa statistic, whilst the overall scale used the intraclass correlation coefficient statistic. A threshold agreement of ≥0.61 was acceptable.

**Results:**

We received 26 responses and a 72% response rate. The first phase of testing found moderate reliability with intraclass correlation coefficients of 0.46 and 0.55 for the articles. The interrater reliability of the scales varied between and within the researchers (0.35, 0.60) and was more consistent with the postgraduate students (0.54, 0.54). Five items on the scale scored below the threshold and were revised for further testing. In this phase the intraclass correlation coefficient demonstrated variability between articles but improved to achieve reliability above the agreed threshold.

**Conclusion:**

This study provides evidence of the reliability of the NICMAN scale although improvements to a small number of items remain.

## 1. Background

Acupuncture practice has evolved over time and in response to different cultural contexts and today represents a broad range of styles of practice. Acupuncturists commonly use several stimulation techniques and modalities including manual and electroacupuncture, cupping, moxibustion, herbs, tuina, and acupressure. Indeed, acupuncture is a complex intervention. Practice styles vary, and the skill and expertise of the practitioner may influence the outcome of treatment. To increase transparency and the reproducibility of acupuncture performed in clinical trials, many acupuncture interventions use standardised or semistandardised treatments and standardised intervention protocols.

There are now clear standards for publications to report acupuncture parameters used in clinical studies [1], and the time is right to address the related but separate issue of evaluating the quality of acupuncture performed during these studies. Ensuring adequate reporting of a study may contribute to improving its reliability but does not address the quality of intervention applied (i.e., validity, standards, or adequacy).

An assessment of quality can be based on conceptual and operationalized definitions of what quality means and how to measure it. Measurement may involve the development of standards, generally derived from two sources [2]. Empirical standards can be derived from actual practice and are used to compare care in one setting with that in another setting. Alternatively, normative standards are derived from sources that set standards of knowledge (e.g., by standard textbooks, publications, panels of practitioners, or research staff in consultation with practitioners) [3].

In 2008, funding was received by the National Institute of Complementary Medicine (NICM) to establish a network of acupuncture researchers in Australia, the National Institute for Complementary Medicine Acupuncture Network (NICMAN), and for the network to undertake the development of an instrument to assess the quality acupuncture administered in clinical research. In 2011 we reported on the initial development of the instrument, the NICMAN scale, and the Delphi process used to achieve consensus on defining the domains and items relating to the acupuncture administered within a clinical trial context [4]. The scale comprised 14 domains and 26 items relating to quality acupuncture. The domains related to study design, rationale of the intervention, and specific criteria relating to the acupuncture characteristics including needling stimulation whether manually or using electrostimulation, duration, and frequency of treatment and practitioner training.

Scales to evaluate the methodological quality of studies have generally not undergone extensive validity and reliability testing [5, 6]. During the early developmental work phase, concurrent and content validity was established [4]. The items included in the NICMAN scale were derived from a Delphi consensus process [7] and therefore demonstrate face validity. Work on establishing the convergent validity and reliability of the scale remained. Reliability includes elements such as internal consistency (intrarater), including an assessment of agreement of scores between different assessors (interrater).

Convergent validity examines the extent to which scores of a particular instrument correlate with another measure using the same construct. However, an existing measure to assess the quality of acupuncture included only four questions relating to the choice of acupuncture points, the number of sessions and duration, needling technique, and acupuncturist's experience. These items were then rated as adequate, inadequate, and do not know [8]. The NICMAN scale however includes additional constructs and it was considered inappropriate to proceed with using the quality of acupuncture scale to establish the convergent validity of the NICMAN scale.

Following publication of the NICMAN scale we reviewed the scale and made revisions combining and reducing the domains to 11 and 16 items. We also assigned weightings to individual items and the calculation of a total score. The majority of scales that have been constructed use scoring of 1, 2, and 3 thresholds [9]. The weightings assigned to the 11 domains reflected the opinions of the working group (CS, AB, SC, and XZ) that not all items have the same relationship to defining quality. We allocated scores as follows: 2 points for yes, 0 for no, and 1 for unclear or partial agreement. The responses to the individual items are summed to create an overall summary score representing the quality of the acupuncture administered. The scale retained instructions on how to interpret the scale and assign a score. The aim of this study was to examine the reliability of the scale across the two participant groups and to elicit participants' views of the instrument and its degree of coherence with their judgement of the acupuncture research papers.

## 2. Materials and Methods

This research was approved by the Western Sydney University Human Ethics Committee (H10834).

### 2.1. Participants

We anticipate the finalised NICMAN instrument would be used by academics and students alike to critically appraise the quality of acupuncture studies. Therefore it was considered important that the tool could be used by both groups who may have different research literacy capabilities. Two populations were involved with testing the reliability of the instrument. We invited the 15 acupuncture researchers in the acupuncture network from the three Australian universities. Secondly, 21 postgraduate coursework students enrolled in the Masters of Health Science (Traditional Chinese Medicine) at Western Sydney University during 2014-15 were approached and invited to participate.

#### 2.1.1. Study Implementation

Participants were sent an email containing a copy of a participant information sheet explaining the purpose of the study and an electronic link to the instrument hosted on SurveyMonkey (https://www.surveymonkey.com). Participants were asked to rate two articles using the scale [10, 11]. In addition we included additional open ended questions examining their views on the criteria used to assess acupuncture quality, the appropriateness of the response choices, the use of the rating criteria “unclear” or “partial” within the same score, and their views about the strengths, weaknesses, or usefulness of the NICMAN scale when applying it in practice and whether the total score on the scale fitted with their overall judgement of the quality of the papers.

Following this stage of testing, modifications were made to scale items and the exercise was repeated using an additional two research reports [12, 13]. Reliability was reassessed using the acupuncture research group only.

#### 2.1.2. Sample Size and Analysis

Interrater reliability of the individual scale items was assessed using the Fleiss kappa statistic which can be used to rate data by multiple raters [14]. A weighted kappa was not applied to individual items due to the absence of a method to calculate the statistic for multiple raters. The kappa statistic treats the ordinal data as nominal and describes agreement between observers as 0 for chance agreement only and 1 for perfect agreement. It has been described within six categories, <0 less than chance agreement, 0.01–0.20 slight agreement, 0.21–0.40 fair agreement, 0.41–0.60 moderate agreement, 0.61–0.80 substantial agreement, and 0.81–0.99 almost perfect agreement [15]. The total of the 16 items produces a score in the range 0–33. Intraclass correlation (which is mathematically identical to weighted kappa with linear weights [16]) is used to summarise the reliability of this numeric total. For interpretation, the same six-category system can be applied [17]. The ICC could not be used for the individual items, due to a limited number of categories.

When considering sample size, the number of items is fixed at 16 items but the number of raters can vary. We anticipate substantial agreement or better (i.e., ≥0.61) and sought to keep the lower bound of the 95% confidence interval for ICC within the substantial agreement category or close to it. Following Zou 2012 [17] a sample size of 25 raters on 16 items would have 80% power to keep the lower bound of the confidence interval within 20 percentage points (one category) of the observed ICC. That is, the lower bound should never be less than the moderate agreement.

The initial analyses examined the reliability of the scale for both groups. To calculate the kappa statistic we used the online programme http://justusrandolph.net/kappa/. To calculate the ICC we used SPSS [18] and reported 95% confidence intervals (CI). We accepted substantial agreement or better (≥0.61). For items of lower agreement we explored if there were differences between the two groups. We also examined the ease of use and reliability among experienced researchers and postgraduate course work acupuncture students. A descriptive analysis was made of the open text responses, with deidentified quotations included to illustrate participant's views about the scales.

## 3. Results

Responses to the survey were received in February 2015. We received 10 responses from acupuncture researchers and 16 responses from the Masters students, giving a 72% response rate.

We observed that items (6a) and (6b) had 20% and 50% missing data, respectively. To avoid losing all data from the majority of raters we have excluded these two items from the calculation of the ICC. Even after the exclusion of items (6a) and (6b) we found only moderate reliability in the coding of both papers with ICCs of 0.46 and 0.55 for papers 1 and 2, respectively. In paper 1 six items were rated as almost perfect agreement using the kappa statistic, two items were rated as substantial agreement, four items had moderate agreement, and four items scored fair (Table 1). For paper 2, three items scored almost perfect, four items had substantial reliability, three items were rated as moderate, and six items were rated as fair (Table 1). Items with consistently higher kappa reliability for both papers included items one to four describing the study design population, intervention, comparator, and outcome, item (5) study design appropriate to question, and item (10) treatment numbers for a chronic condition.

After excluding items (6a) and (6b), the interrater reliability of the scales was found to be consistent for the postgraduate students with an ICC of 0.54 (95% CI 0.21 to 0.80) on paper 1 and 0.54 (95% CI 0.24 to 0.80) on paper 2. The acupuncture researchers displayed greater variability with an ICC of 0.35 (95% CI −0.06 to 0.76) on paper 1 and 0.60 (95% CI 0.29 to 0.86) for paper 2.

Overall we identified five items (items (6a), (6b), (7a), (7a), and (11)) which scored consistently below the threshold score of 0.61 reliability in both paper one and two (Table 1).

## 4. Participants' Views on the NICMAN Scale

Overall the feedback obtained from both groups of participants was positive. The majority of respondents were comfortable with the relevance of the criteria to assess acupuncture research quality. Specific remarks were made in response to how the scale or criteria could be improved. Comments included that there was no question “about masking, outcome measures, follow up.” Another commented that “I believe that there was doubling up on questions relating to descriptions of interventions giving it a disproportionate skew on final number.” Participants reported they found the choices on which to judge the criteria appropriately. A suggestion was made to use a sliding scale and that at times it was “hard to differentiate between unclear and partial.” This comment reflects the findings of the assessment of item quality where different interpretation appears to have been impacted by the quality of study reporting.

We were also interested to examine whether the individual scoring of items and total score scale matched an overall impression of study quality. Twenty participants (77%) considered the score did meet their assessment of the quality of the papers. Some respondents were unequivocal that the final score confirmed their reading of the papers. One participant welcomed the scale as “it will encourage researchers to plan better acupuncture protocol and delivery [if it is] attach[ed] to CONSORT Statement” and another stated that it is an “appropriate time for this sort of scale.” Another participant commented that “two terms have similar meanings in most cases so current approach ok. More options make it difficult to choose.”

Fourteen participants (54%) viewed the scale as a positive contribution to acupuncture research. Three (12%) respondents reported a categorical “no” in response to this question, and 5 participants qualified their support for the scale. These qualifications included specific comments on the scale relating to clarification of individual items within a domain or the entire domain. Sixteen participants (62%) indicated that applying the scale changed their evaluation of the acupuncture research articles as they read each paper. One participant reported they considered the scale positive “in relation to closeness of replication to actual TCM practice.” Others reported that the scale was “only looking at intervention, not the control or study quality or qualitative aspects with a study design.” Another participant reported “at points in both papers I was thinking ‘that's good - it is described' and was being lenient and then at other points the scale helped focus.”

## 5. Additional Phase of Testing

Following a review of the initial testing phase, items scoring below our reliability threshold were identified ((6a), (6b), (7a), (7b), and (11)) and considered for revisions and further testing. It was decided to retain item (6a) unchanged. Item (6b) was not changed but combined within item (11) to read (a)* the acupuncturist administering the intervention is registered with a regulatory authority or meets at least the minimum WHO standard [19], and (b) the practitioner undertaking the TCM differential diagnosis is adequately trained, for example, registered with a regulatory authority, or meets at least the minimum WHO standard [19].*

Items (7a) and (7b) were replaced with a statement to read “acupuncture points selected are consistent with chosen treatment principles. A statement is provided stating the acupuncture prescription is consistent with literature review, expert opinion or text books.”

We then repeated the exercise of rating two other articles [12, 13] with the acupuncture researcher group only (Table 2). The ICC was consistent with previous ratings for paper 3 (ICC 0.68, 95% CI 0.37 to 0.98) but lower for paper 4 (ICC −0.19, 95% CI −0.45 to 0.29). Item (6a) was left unchanged* (differential diagnosis (if undertaken) is stated)* but retested and demonstrated higher reliability in paper 3 but not paper 4. Following testing of the revised* item (7) *there was some improvement but variation to 0.35 and 0.56 for item (7) (Table 2). The quality of the practitioner was assessed previously in item (6) (0.25, 0.37, Table 1) and now combined with item (11) demonstrated significantly improved reliability with kappa statistics of 1.0 in one paper (paper 4) but variation again in paper 3 with reliability of 0.31.

## 6. Discussion

We evaluated the reliability of the NICMAN scale across two participant groups using a total of four papers reporting on acupuncture clinical trials. The reliability was initially moderate, improving to substantial when the modified tool was applied to paper 3. The modified tool applied to paper 4 resulted in complete agreement on (6) of (11) items. However, as the ICC is a measure of variance explained, the absence of variation meant that these (6) items did not contribute to the ICC undermining the overall ICC accordingly. We were unable to calculate a weighted measure of reliability for the individual items, and therefore for the individual item kappa statistics do not reflect the ordinal nature of the scale. We had to exclude the poor performing items (6a) and (6b) from the overall reliability scores for papers 1 and 2 but were able to include these items during evaluation of the modified scale. The inclusion of open questions provided additional feedback supporting the perception of overall quality and a useful contribution to acupuncture research.

The need to address the quality of acupuncture interventions in clinical trials has been highlighted over several decades and increasingly so when reviewing and appraising systemic reviews of acupuncture [20]. The assessment of the quality of acupuncture administered in RCTs and systematic reviews has been problematic, and although several attempts have been made, developments have stalled due to poor reliability during the development and testing phases. The assessment of the reliability of the NICMAN scale experienced similar methodological challenges, but with refinement we propose that this finalised version (Table 3) demonstrates acceptable reliability for wider use. Our findings may also be influenced by the wording of individual statements, interpretation of the questions, and scoring instructions and additional explanatory materials may assist with further reducing the potential for differences in the interpretation of statements. Furthermore we recognise that the scale may have the potential to demonstrate better reliability if there is greater adherence with the reporting standards for randomised controlled trials detailed in the CONSORT Statement [2]. Reporting quality that clearly describes the information about the design, conduct, and analysis of the trial makes the interpretation of studies and assessment easier. A further limitation of our study relates to the precision of the NICMAN scale that also needs to be considered. Few items had perfect reliability, although the majority reached our threshold reliability score and we have demonstrated that testing of the scale in two phases displayed moderate agreement.

The participants were acupuncture researchers and postgraduate students undertaking course work, and we found some variation in the reliability scores, with greater variability found among the researchers. It is unclear if this difference is influenced by interpretation of the manuscripts or skills in rating and appraising the literature.

We propose that the scale is not used alone, but to be included as part of a methodological appraisal of individual acupuncture RCTs using common critical appraisal tools, and could be applied when also undertaking systematic reviews. Used together these instruments could provide a comprehensive assessment of the methodology of the study design and the quality of the acupuncture intervention being evaluated. The approach taken in developing the NICMAN scale also provides a template for the development of other ancillary approaches such as moxibustion, cupping, and scrapping* (guasha)*.

## 7. Conclusion

This study provides evidence of the reliability of the NICMAN scale although improvements to a small number of items remain as well as the need for robust explanatory documentation to accompany the scale.

## Figures and Tables

**Table 1:** Kappa statistic and intraclass correlation coefficient for all participants rating two papers.

Items	Paper 1 kappa statistic	Paper 2 kappa statistic
(1) Clearly described population	0.66	0.85

(2) Clearly described intervention	0.92	0.79

(3) Clearly described comparator	0.85	0.85

(4) Clearly described outcome	0.92	0.68

(5) Study design appropriate to question	0.72	0.73

(6a) Differential diagnosis (if undertaken) is stated	0.37	0.25

(6b) The practitioner undertaking the differential diagnosis is adequately trained, for example, registered with a regulatory authority, or meets at least the minimum WHO standard	0.35	0.60

(7a) Acupuncture points are selected according to the diagnosis in item (6)	0.40	0.32

(7b) The rationale for the acupuncture points is sourced from expert opinion, literature review, or text books	0.42	0.42

(8) Point location is described in published standard acupuncture location texts used as a reference, or location described in anatomical terms and/or an accurate proportional method for locating acupoints is used	0.31	0.92

(9a) Needle brand and dimension are used consistently across all participants and sessions	1.00	0.31

(9b) Depth of needle insertion is reported and referenced to a standard text or mm or range is stated	0.52	0.46

(9c) Manual needle manipulation is justified (in the absence of needle manipulation justification is provided for the decision not to undertake needle manipulation). If applicable, electroacupuncture device should be identified and approved in country of use	0.51	0.31

(9d) Needle sensation was sought and described	1.00	0.37

(10) If it is a chronic condition a minimum of six treatments are administered; if fewer treatments are delivered appropriate justification is documented	0.92	0.78

(11) The acupuncturist administering the intervention is registered with a regulatory authority or meets at least the minimum WHO standard	0.47	0.41

*ICC for overall agreement (95% CI) excluding items (6a) and (6b)*	*0.46* *(0.19 to 0.70)*	*0.55* *(0.32 to 0.75)*

For *paper 1*, see [10]; for *paper 2*, see [11].

**Table 2:** Phase two revised Scale kappa statistics and intraclass correlation coefficient for NICMAN participants rating two papers.

Item number	Paper 3 kappa statistic	Paper 4 kappa statistic
(1) Clearly described population	0.82	1.0

(2) Clearly described intervention	0.82	1.0

(3) Clearly described comparator	1.0	1.0

(4) Clearly described outcome	0.82	1.0

(5) The study design is appropriate for the research question	0.31	0.56

(6) A differential diagnosis (if undertaken) is stated	0.65	0.27

(7) Acupuncture points selected are consistent with chosen treatment principles. A statement is provided stating the acupuncture prescription is consistent with literature review, expert opinion, or text books	0.35	0.56

(8) Needling: depth and manipulation (a) Needle brand and dimension are used consistently across all participants and sessions. We use the term “consistently” to provide an aspect relating to quality rather than focus on reporting (b) Depth of needle insertion is reported and referenced to a standard text or mm or range is stated (c) Needle manipulation is justified (in the absence of needle manipulation justification is provided for the decision not to undertake needle manipulation) (d) Needle sensation was sought and described (e) Electroacupuncture device should be identified and approved in country of use	0.35	0.56

(9) Point location: (a) published standard acupuncture location texts are used as a reference, or (b) location described in anatomical terms and/or an accurate proportional method for locating acupoints is used	0.35	0.31

(10) Number of treatments (a) If it is a chronic condition a minimum of six treatments are administered; if fewer treatments are delivered appropriate justification is documented (b) If it is an acute or subacute condition no minimum number of treatments are specified, but justification is to be provided	0.82	1.0

(11) (a) The acupuncturist administering the intervention is registered with a regulatory authority or meets at least the minimum WHO standard [19] (b) The practitioner undertaking the TCM differential diagnosis is adequately trained, for example, registered with a regulatory authority, or meets at least the minimum WHO standard [19] (if no differential diagnosis √NA)	0.31	1.0

*ICC for overall agreement (95% CI)*	*0.68* *0.37 to 0.89*	*−0.19* *−0.45 to 0.29*

For *paper 3*, see [13]; for *paper 4*, see [12].

**Table 3:** Final NICMAN scale.

	Item Author of study Person extracting the date	*Yes*: the item is adequately *reported/described* and *meets a satisfactory standard*	*No*: the item is adequately *reported/described* but does *not meet a satisfactory standard*	*Unclear*: the item is *partially or not reported/described* and you are *unable to make a judgement or* you partially agree with the response to the item	*Not applicable*: the item may meet the nonapplicable criteria if the study item is not relevant (full score still given, only applied to some items)
(1)	Clearly described population	2	0	1	

(2)	Clearly described intervention	2	0	1	

(3)	Clearly described comparator	2	0	1	

(4)	Clearly described outcome	2	0	1	

(5)	The study design is appropriate for the research question	3	0	1 or 2	

(6)	A differential diagnosis (if undertaken) is stated (2 points for yes, 0 for no, 1 for unclear or partial agreement)	2	0	1	

(7)	Acupuncture points selected are consistent with chosen treatment principles. A statement is provided stating the acupuncture prescription is consistent with literature review, expert opinion, or text books	2	0	1	

(8)	*Needling: depth and manipulation* (i) Needle brand and dimension are used *consistently* across all participants and sessions. We use the term “consistently” to provide an aspect relating to quality rather than focus on reporting (ii) Depth of needle insertion is reported and referenced to a standard text or mm or range is stated (iii) Needle manipulation is justified (in the absence of needle manipulation justification is provided for the decision not to undertake needle manipulation) (iv) Needle sensation was sought and described (v) Electroacupuncture device should be identified and approved in country of use	2	0	1	

(9)	*Point location* (i) Published standard acupuncture location texts are used as a reference, or (ii) location described in anatomical terms and/or an accurate proportional method for locating acupoints is used	2	0	1	

(10)	*Number of treatments* (i) If it is a chronic condition a minimum of six treatments are administered; if fewer treatments are delivered appropriate justification is documented (ii) If it is an acute or subacute condition no minimum of treatments are specified, but appropriate justification is to be provided	2	0	1	

(11)	(i) The acupuncturist administering the intervention is registered with a regulatory authority or meets at least the minimum WHO standard [19] (ii) The practitioner undertaking the TCM differential diagnosis is adequately trained, for example, registered with a regulatory authority, or meets at least the minimum WHO standard [19] (if no differential diagnosis √NA)	2	0	1	

Total score					

## References

[1] MacPherson H., Altman D. G., Hammerschlag R. (2010). Revised standards for reporting interventions in clinical trials of acupuncture (STRICTA): extending the consort statement. *Acupuncture in Medicine*.

[2] Prady S. L., Richmond S. J., Morton V. M., MacPherson H. (2008). A systematic evaluation of the impact of STRICTA and CONSORT recommendations on quality of reporting for acupuncture trials. *PLoS ONE*.

[3] Donabedian A. (2005). Evaluating the quality of medical care. *Milbank Quarterly*.

[4] Smith C. A., Zaslawski C. J., Zheng Z. (2011). Development of an instrument to assess the quality of acupuncture: results from a Delphi process. *Journal of Alternative and Complementary Medicine*.

[5] Jadad A. R., Moore R. A., Carroll D. (1996). Assessing the quality of reports of randomized clinical trials: is blinding necessary?. *Controlled Clinical Trials*.

[6] Olivo S. A., Macedo L. G., Gadotti I. C., Fuentes J., Stanton T., Magee D. J. (2008). Scales to assess the quality of randomized controlled trials: a systematic review. *Physical Therapy*.

[7] Adler M., Ziglio E. (1996). *Gazing into The Oracle: The Delphi Method and Its Application to Social Policy and Public Health*.

[8] Furlan A., van Tudler M. W., Cherkin D. C., Tsukayama H., Lao L., Koes B. W. (2005). Acupuncture and dry needling for low back pain. *The Cochrane Database of Systematic Reviews*.

[9] Panagiotakos D. (2009). Health measurement scales: methodological issues. *Open Cardiovascular Medicine Journal*.

[10] Mao J. J., Bruner D. W., Stricker C. (2009). Feasibility trial of electroacupuncture for aromatase inhibitor—related arthralgia in breast cancer survivors. *Integrative Cancer Therapies*.

[11] Mehta P. K., Polk D. M., Zhang X. (2014). A randomized controlled trial of acupuncture in stable ischemic heart disease patients. *International Journal of Cardiology*.

[12] Berman B. M., Lao L., Langenberg P., Lee W. L., Gilpin A. M., Hochberg M. C. (2004). Effectiveness of acupuncture as adjunctive therapy in osteoarthritis of the knee. *Annals of Internal Medicine*.

[13] Zhang W.-J., Yang X.-B., Zhong B.-L. (2009). Combination of acupuncture and fluoxetine for depression: a randomized, double-blind, sham-controlled trial. *Journal of Alternative and Complementary Medicine*.

[14] Fleiss J. L. (1971). Measuring nominal scale agreement among many raters. *Psychological Bulletin*.

[15] Landis J. R., Koch G. G. (1977). The measurement of observer agreement for categorical data. *Biometrics*.

[16] Fleiss J. L., Cohen J. (1973). The equivalence of the weighted kappa and the intraclass correlation coefficient as measures of reliability. *Educational and Psychological Measurement*.

[17] Zou G. Y. (2012). Sample size formulas for estimating intraclass correlation coefficients with precision and assurance. *Statistics in Medicine*.

[18] IBM Corp (2013). *IBM SPSS Statistics for Windows*.

[19] WHO Traditional Medicine Unit (1999). *Guidelines on Basic Training and Safety in Acupuncture*.

[20] White A., Cummings M., Barlas P. (2008). Defining an adequate dose of acupuncture using a neurophysiological approach—A narrative review of the literature. *Acupuncture in Medicine*.

